# Unilateral, 3D Arm Movement Kinematics Are Encoded in Ipsilateral Human Cortex

**DOI:** 10.1523/JNEUROSCI.0015-18.2018

**Published:** 2018-11-21

**Authors:** David T. Bundy, Nicholas Szrama, Mrinal Pahwa, Eric C. Leuthardt

**Affiliations:** ^1^Department of Biomedical Engineering,; ^2^Department of Anatomy and Neurobiology,; ^3^Department of Neurological Surgery,; ^4^Center for Innovation in Neuroscience and Technology, and; ^5^Department of Mechanical Engineering and Materials Science, Washington University, St. Louis, Missouri 63130

**Keywords:** BCI, ECoG, electrocorticography, ipsilateral, reach

## Abstract

There is increasing evidence that the hemisphere ipsilateral to a moving limb plays a role in planning and executing movements. However, the exact relationship between cortical activity and ipsilateral limb movements is uncertain. We sought to determine whether 3D arm movement kinematics (speed, velocity, and position) could be decoded from cortical signals recorded from the hemisphere ipsilateral to the moving limb. By having invasively monitored patients perform unilateral reaches with each arm, we also compared the encoding of contralateral and ipsilateral limb kinematics from a single cortical hemisphere. In four motor-intact human patients (three male, one female) implanted with electrocorticography electrodes for localization of their epileptic foci, we decoded 3D movement kinematics of both arms with accuracies above chance. Surprisingly, the spatial and spectral encoding of contralateral and ipsilateral limb kinematics was similar, enabling cross-prediction of kinematics between arms. These results clarify our understanding that the ipsilateral hemisphere robustly contributes to motor execution and supports that the information of complex movements is more bihemispherically represented in humans than has been previously understood.

**SIGNIFICANCE STATEMENT** Although limb movements are traditionally understood to be driven by the cortical hemisphere contralateral to a moving limb, movement-related neural activity has also been found in the ipsilateral hemisphere. This study provides the first demonstration that 3D arm movement kinematics can be decoded from human electrocorticographic signals ipsilateral to the moving limb. Surprisingly, the spatial and spectral encoding of contralateral and ipsilateral limb kinematics was similar. The finding that specific kinematics are encoded in the ipsilateral hemisphere demonstrates that the ipsilateral hemisphere contributes to the execution of unilateral limb movements, improving our understanding of motor control. Additionally, the bihemisheric representation of voluntary movements has implications for the development of neuroprosthetic systems for reaching and for neurorehabilitation strategies following cortical injuries.

## Introduction

Although we traditionally understand voluntary motor movements to stem from the cortex within the hemisphere contralateral to a moving limb, there is increasing evidence that the ipsilateral hemisphere also plays an active role in the execution of voluntary motor movements. Across a variety of modalities in both human subjects and animal models, ipsilateral cortical activations have been observed during unilateral limb movements (Tanji et al., 1988; Aizawa et al., 1990; Wisneski et al., 2008; Ganguly et al., 2009; Buetefisch et al., 2014; Hotson et al., 2014). Similarly, ipsilesional motor deficits have been observed in human patients following unilateral cortical injuries (Baskett et al., 1996; Sunderland, 2000; Schaefer et al., 2007, 2009a,b, 2012). Although this evidence supports the idea that the ipsilateral hemisphere may be involved in the execution of voluntary motor movements, the exact role of the ipsilateral hemisphere remains uncertain.

Defining how the brain encodes motor kinematics (i.e., speed, velocity, and position) is essential to understanding the cortical dynamics that underpin motor control in humans. With regards to ipsilateral motor activations, previous studies have demonstrated that some limited movement kinematics can be decoded from the ipsilateral hemisphere (Ganguly et al., 2009; Hotson et al., 2012, 2014). The extent and detail of the information that is encoded ipsilateral to a moving limb, however, is currently unknown. Additionally, how this ipsilateral kinematic information compares to contralateral kinematic encoding also remains largely unresolved because previous studies have reached conflicting conclusions. Some investigators found that ipsilateral motor activations occur at distinct frequencies (35–50 Hz) and locations (premotor) relative to contralateral motor activations (Wisneski et al., 2008), whereas others have found that contralateral and ipsilateral movement-related activations are similar (Fujiwara et al., 2017; Haar et al., 2017). These discrepant results underscore the need to define, to the highest level possible, how a given hemisphere represents multidimensional ipsilateral and contralateral kinematics. Specifically, encoding detailed movement parameters such as kinematics, joint angles, or muscle activations is a necessary condition for a given hemisphere to play a role in planning and executing voluntary motor movements. Further, understanding the link between cortical physiology and movement is essential to crafting more informed rehabilitation strategies in the setting of brain injuries and movement disorders.

**Figure 1.**
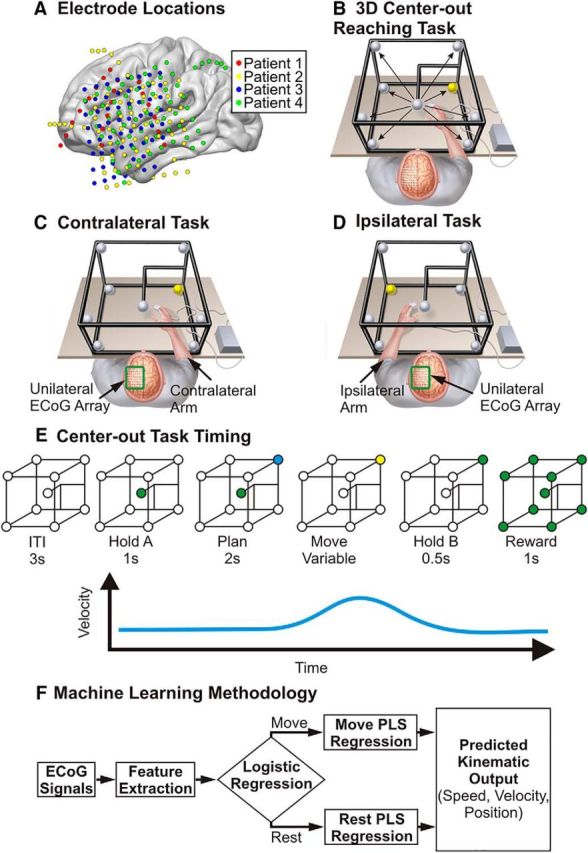
Study methodology. Patients implanted with electrocorticography arrays completed a 3D center-out reaching task. ***A***, Electrode locations were based upon the clinical requirements of each patient and were localized to an atlas brain for display. ***B***, Patients were seated in the semirecumbent position and completed reaching movements from the center to the corners of a 50 cm physical cube based upon cues from LED lights located at each target while hand positions and ECoG signals were simultaneously recorded. Each patient was implanted with electrodes in a single cortical hemisphere and performed the task with the arm contralateral (***C***) and ipsilateral (***D***) to the electrode array in separate recording sessions. ***E***, The task incorporated a center hold period (Hold-A), planning delay, movement period, and exterior hold period (Hold-B). To decode kinematics of contralateral and ipsilateral reaching movements, a hierarchical PLS regression that incorporated a logistic regression classification of movement and rest periods to switch the predicted output between the output of two PLS regression models was used. The first PLS model was trained using data from the rest periods to predict speed and velocity during rest periods and the second PLS regression model was trained using data from the movement periods to predict speed and velocity during movement periods (***F***). (***E*** adapted from Bundy et al., 2016 under terms of the CC BY license).

In this study, motor-intact humans implanted with unilateral intracranial electrocorticographic (ECoG) electrodes performed a 3D center-out reaching task with each arm (Fig. 1*B–D*). We initially hypothesized that unilateral ECoG signals would enable us to decode movement kinematics of both limbs with distinct features distinguishing each arm. We found that ECoG signals could be used to decode 3D kinematics of ipsilateral limb movements. Additionally, we found that ECoG representations of reaching movements are conserved between contralateral and ipsilateral limb movements. Together, these findings support that the ipsilateral hemisphere plays an active role in unilateral arm movement and that kinematic information has substantial bihemispheric representation.

## Materials and Methods

### 

#### 

##### Experimental design and statistical analyses.

The experiments presented used within-subject designs. As described in detail below, the statistical significance of movement-related neural activity was tested using a one-sample *t* test after *z*-scoring data relative to baseline periods. Multiple comparisons across electrodes, neural features, time windows, and task condition were controlled for using false discovery rate correction (Benjamini and Yekutieli, 2001). The statistical significance of kinematic prediction accuracies for individual patients as well as across patients and cross folds was evaluated with a Wilcoxon rank sum test comparing actual and surrogate prediction accuracies with Bonferroni correction performed to control for multiple comparisons. Finally, the similarity of the absolute value of activation patterns for the contralateral and ipsilateral models was evaluated using Pearson's rho.

##### Patient population.

The study included four patients with intractable epilepsy undergoing temporary placement of subdural ECoG electrodes for localization of their epileptic foci. Electrodes were implanted for ∼1 week (7–14 d). We previously described a method to predict kinematics of contralateral arm reaching movements in five patients (Bundy et al., 2016). A subset of four patients (three male, one female) from our earlier study performed a 3D reaching task both with the limb contralateral to their electrode array as well as the limb ipsilateral to the electrode array in separate recording sessions. ECoG electrodes were located in the hemisphere contralateral to the dominant hand in all patients. One patient (Patient 2) described feeling minor pain and fatigue in the arm contralateral to the electrode array due to mass effects from the implant (i.e., motor weakness caused by the electrode array physically pressing against the motor cortex), although this did not lead to any observed functional deficits. Table 1 describes patient characteristics for each patient. The Institutional Review Board of the Washington University School of Medicine approved the study protocol and all patients provided written informed consent before participating in the study.

**Table 1.** Patient characteristics and task performance

Patient number	Electrode locations	Epileptic focus	Handedness	Age (y)	Contralateral trials	Ipsilateral trials
1	Right temporal/frontal strips	Right mesial temporal	Left	40	288	128
2	Left frontotemporal grid and strips	Left anterior/mesial Temporal	Right	27	104	240
3	Left frontotemporal grid and strips	Left frontal/central	Right	18	256	256
4	Left frontotemporal grid and strips	Left anterior sub-temporal	Right	56	256	256

##### Reaching task.

Each patient performed a 3D center-out reaching task. The task has been described in detail previously (Bundy et al., 2016). In short, hand positions for the moving limb were collected using a Flock of Birds motion capture system (Ascension Technology, Shelburne, VT). A single sensor was fixed to the top of the index and middle finger to track hand position. Hand position was synchronized with ECoG signals through a custom BCI2000 module. The center-out task consisted of reaches to eight targets at the corners of a physical cube with 50-cm-long edges. Patients were seated in a semirecumbent position and the center target was placed at their midline ∼40 cm in front of their chest (Fig. 1*B*). Each trial of the task included a cue to move their hand to a center target position, a baseline period with their hand remaining at the central target (Hold-A, 1 s), a planning delay period (2 s), a movement period, and finally an outer hold (Hold-B, 0.5 s) period. All task cues were visual cues provided by colored LED lights at the center and peripheral target locations. To compare contralateral and ipsilateral arm movements, the task was first performed using the arm contralateral to the electrode array in one session followed by a second session in which patients used the arm ipsilateral to the electrode array (Fig. 1*C*,*D*). In three of the four patients (Patient 1, Patient 3, and Patient 4), contralateral and ipsilateral movement sessions were performed on different days.

##### Data acquisition.

Each patient was implanted with subdural platinum–iridium ECoG grid and strip electrodes (PMT or Ad-Tech) with an electrode diameter of 4 mm (2.3 mm exposed) and an electrode spacing of 1 cm. The number and locations of electrodes were solely based upon the clinical requirements for the localization of each patient's epileptic focus; however, each patient had some coverage of their motor cortex. A subdural 1 × 4 or 1 × 6 strip of electrodes was also implanted facing the skull for use as ground and reference signals. Signals were sampled at 1200 Hz and recorded using g.USBamp biosignal amplifiers (g.tec) and the BCI2000 software system (RRID:SCR_007346) (Schalk et al., 2004). No external filters were used with the exception of the amplifier's internal anti-aliasing filter. The g.USB amplifiers used had an internal sampling frequency of 38.4 kHz and an internal antialiasing filter at 5 kHz.

##### Electrode localization.

Electrode locations were estimated from lateral radiographs collected after electrode implantation. The getLOC package (Miller et al., 2007) was used to localize electrodes onto an atlas brain with an accuracy of ∼1 cm and are displayed in Figure 1*A*. To display the topographic organization of signal features, we mapped quantitative results onto an atlas brain using a weighted spherical Gaussian kernel centered at each electrode location. Gaussian kernels from all electrodes were linearly superimposed and the contribution from each electrode was normalized based upon the number of nearby electrodes.

##### ECoG processing.

A detailed description of the processing sequence is contained in our previous study (Bundy et al., 2016). Initially, ECoG signals were visually inspected in the time and frequency domain and channels displaying nonphysiologic activity or pathological epileptic activity throughout a recording were excluded (Patient 1: 7 electrodes, Patient 2: 7 electrodes, Patient 3: 4 electrodes, Patient 4: 19 electrodes). Additionally, ECoG data from each individual trial was examined in the time and frequency domain and kinematic data from each trial was visually examined to exclude trials with significant artifacts or kinematic data outside of the sampling range of the motion capture system. After this process, the total number of trials analyzed for each patient was as follows: Patient 1: 245 contralateral, 119 ipsilateral; Patient 2: 76 contralateral, 187 ipsilateral; Patient 3: 221 contralateral, 177 ipsilateral; Patient 4: 202 contralateral, 208 ipsilateral. ECoG signals were then re-referenced to the common average of each array or amplifier, band-pass filtered between 0.1 and 260 Hz, and notch filtered to remove all noise harmonics <260 Hz.

Because of their relevance in previous studies of movement decoding (Schalk et al., 2007; Pistohl et al., 2008; Hotson et al., 2014), spectral power changes and the local motor potential (LMP) were then calculated. The maximum entropy method, an autoregressive spectral estimation method, was used to estimate the spectral power of the ECoG signals (Marple, 1987). A model order of 75 was chosen and spectral power was estimated in 2 Hz frequency bins with bin centers from 3 to 253 Hz. To examine temporal changes in spectral power, spectral power was calculated in 300 ms windows with shifts of 50 ms between windows. Power spectra were normalized using a log transform and were then converted to *z*-score values relative to the period from 200 ms after the beginning of the baseline Hold-A period until the end of the Hold-A period. Spectral power was then pooled within seven canonical frequency bands that spanned relevant frequency bands while avoiding noise harmonics. Specifically, the bands used were as follows: theta (4–8 Hz), mu (8–12 Hz), beta 1 (12–24 Hz), beta 2 (24–34 Hz), gamma 1 (34–55 Hz), gamma 2 (65–95 Hz), and gamma 3 (130–175 Hz). Finally, the band-averaged power was once again *z*-scored with respect to the period from 200 ms after the beginning of the Hold-A period until the end of the Hold-A period. The use of two distinct *z*-score calculations corrected for the 1/f fall-off in spectral power to ensure that each 2 Hz bin contributed equally to each frequency band and that the number of 2 Hz frequency bins averaged into the respective frequency bands did not affect the amplitude or variance of each frequency band. The LMP was calculated by using a second-order Savitzky–Golay smoothing filter on 300 ms time windows shifted by 50 ms between windows. As with the spectral power features, the LMP time series were *z*-scored with respect to the period from 200 ms after the onset of the Hold-A period to the end of the Hold-A period. Although alternative methods for spectral power estimation, such as a wavelet convolution, may have led to improved temporal resolutions, particularly for higher frequencies, the autoregressive method with identical time windows across frequency bands was chosen to directly compare the encoding of movement parameters within each frequency band. Additionally, the availability of a real-time MEM module within BCI2000 allows for decoding models developed here to be applied in potential future online brain–computer interface tasks.

##### Kinematic data processing.

A Flock of Birds motion capture system was used to record 3D hand positions with the positive *x*-axis oriented toward the patient along the anterior–posterior axis, the positive *y*-axis oriented laterally toward the patient's left, and the positive *z*-axis oriented downward along the superior–inferior axis. In addition to hand position, the 3D position values were differentiated to calculate the velocity components in each dimension and these velocity components were also normalized to calculate hand speed. To align kinematic information with ECoG activity, each kinematic parameter was averaged within 300 ms time windows shifted by 50 ms between windows. The onset of movement in each trial was determined as the first time that hand speed exceeded 10% of the trial-specific maximum speed. The threshold of 10% was chosen because the peak hand speed during the premovement planning period did not exceed this threshold in over 75% of trials, providing good separation between movement and rest. The movement onset time was also visually confirmed in all trials and, if needed, corrected to ensure that movement onsets were not aligned to spurious fluctuations in the measured hand positions.

##### Neural correlates of reaching movements.

To examine the movement-related differences in the timing, sign, and amplitude of changes in ECoG activity during reaches of the contralateral and ipsilateral arms, time courses of *z*-scored ECoG features [spectral power in 7 canonical frequency bands: (theta (4–8 Hz), mu (8–12 Hz), beta 1 (12–24 Hz), beta 2 (24–34 Hz), gamma 1 (34–55 Hz), gamma 2 (65–95 Hz), and gamma 3 (130–175 Hz), and LMP amplitude] were aligned and stacked from 1 s before movement onset to 2 s after movement onset for each electrode and feature. Because *z*-score values were previously calculated (see “ECoG processing” section) using the activity during the Hold-A (rest) period, a *z*-score value of zero represents the average activity for the Hold-A period. A one-sample *t* test was used to determine whether the mean *z*-score value for a specific electrode, feature, time window, and hand was significantly different from 0. An independent-samples *t* test was used to determine whether the *z*-score values for a specific electrode, feature, and time window were significantly different between hands. The total number of comparisons across electrodes, features, time windows, and hand were corrected for using the Benjamini–Hochberg–Yekutieli method of false discovery rate (FDR) correction that accounts for the correlated *p*-values caused by the overlapping time windows (Benjamini and Hochberg, 1995; Benjamini and Yekutieli, 2001). The locations and timing of significant power changes were compared between contralateral and ipsilateral arm reaches and were summarized by calculating the percentage of electrodes with significant (*p* < 0.05) amplitude changes at each time window and frequency band for contralateral and ipsilateral limb movements. Differences in timing between contralateral and ipsilateral arm movements were quantified by calculating the correlation (Pearson's *r*) between the time courses of the percentage of active electrodes during contralateral arm movements and the percentage of active electrodes during ipsilateral arm movements. Correlations were calculated using time lags ranging from −500 ms (ipsilateral leading) and 500 ms (contralateral leading) and the time lag with the peak correlation was determined. The peak correlation and time lag were calculated separately for each frequency band examined. Additionally, we calculated the percentage of electrodes in which the absolute value of the *z*-scored feature was significantly different between contralateral and ipsilateral hand movements for each frequency band.

##### Machine learning methods.

Next, we investigated whether we could use ECoG signals to decode kinematics of reaching movements of the ipsilateral limb and compared the ability to decode kinematics of ipsilateral and contralateral limb movements. All examinations of the ability to decode kinematics were performed offline. To test the ability to decode kinematics, the datasets for the contralateral and ipsilateral arm movement conditions from each patient were each divided into separate training and testing sets. We generated a training set by randomly sampling 7/8th of the trials and the remaining trials were held out as a test set. For each trial, the period from 2 s before the onset of movement until the end of the trial was used. Separate contralateral and ipsilateral training and testing sets were constructed for each patient by concatenating the time courses of both neural data and kinematics from the randomly selected trials.

The details of our machine learning algorithm for decoding kinematics has been described previously (Bundy et al., 2016). Because the reaching task incorporated periods where patients were required to hold their hand at the center or exterior of the workspace as well as periods where patients made active reaching movements, we used a hierarchical partial-least-squares (PLS) regression (Fig. 1*F*) that used a logistic regression model to distinguish movement and rest periods and to switch the model output between the output of a movement PLS regression model and the output of a rest PLS regression model, producing the final output.

To predict movement and rest periods, training labels were generated using a threshold of 10% of the maximum movement speed. The model was trained using the *z*-scores of eight features (seven frequencies and LMP) at each channel. For each feature, the time lag with the maximum absolute correlation coefficient between the ECoG activity and movement speed was used. The hyperparameter weights associated with the L1 and L2 norms were optimized via sevenfold cross-validation within the 7/8th training set. After optimizing the hyperparameters, the entire training set was used to train the model.

The output from the logistic regression was used to switch between separate movement and rest PLS regression models. The PLS regression model estimates a lower-dimensional latent structure of the input variables that is used to fit the regression to avoid overfitting and account for multicollinearities. For our model, the inputs were the *z*-scored spectral power and LMP amplitude of each channel for every time lag between −1000 and 500 ms in 50 ms steps with negative lags, indicating the amount of time that the neural activity leads the kinematic data. The model outputs were movement speed and 3D movement velocity (Vx, Vy, and Vz). Finally, to enforce the constraint that the magnitude of the predicted velocity vector equals the predicted speed, the predicted velocity vector was normalized and modulated by the predicted speed. As with the logistic regression, 7-fold cross validation was used within the 7/8th training set to determine the number of latent features used for the final model that was trained using the entire training set.

To generate a distribution of movement prediction accuracies, we repeated the process of randomly selecting 7/8ths of the trials as a training set and using the remaining 1/8th of the trials as a test set 100 times for each patient and hand. A separate model was trained for each of the 100 training sets for each patient and hand and accuracy of the full model was calculated by computing the correlation coefficient between the actual and predicted values for each of the four kinematic parameters (speed, Vx, Vy, and Vz) in each of the held-out test sets. Additionally, the predicted velocity vectors were concatenated across time to produce predicted movement trajectories. The percentage of targets hit was then determined by calculating the percentage of trials in which the actual and predicted movement trajectory ended in the same quadrant.

We evaluated whether the model predictions were better than chance using two surrogate predictions. First, we randomly adjusted the temporal relationship between ECoG signals and kinematics by randomly reordering the training set trials, randomly selecting a new trial onset time for each trial, and generating a new time course by wrapping data from the beginning of the trial to the end of the trial. This procedure maintained the autocorrelation structure of the kinematics while randomizing the relationship between ECoG signals and kinematics. For each training and testing set, we trained one model using the original kinematic data and a second model using the surrogate kinematics. Both models were tested using the original test set. Second, we randomly shuffled the channel and frequency assignments of the model weights 100 times and generated test accuracies with the reshuffled weights. The statistical significance of the kinematic prediction models was evaluated using a Wilcoxon rank–sum test to compare the median actual accuracy with both median surrogate accuracies. The statistical significance was evaluated for the entire dataset including all patients as well as for each patient's individual dataset. Bonferroni correction was used to correct for the total number of prediction features tested (215 comparisons: 5 movement parameters (speed, Vx, Vy, Vz, and percentage targets hit) × 2 hands × 2 cross-prediction conditions × 2 surrogate methods, 5 movement parameters compared between hands, 5 movement parameters × 2 hands compared between true and cross-prediction conditions, and 4 patients × 5 movement parameters × 2 hands × 2 cross-prediction conditions × 2 surrogate methods compared for individual patients) with a critical *p*-value of 0.00023 representing a statistically significant result. Importantly, to generate surrogate predictions the actual logistic regression predictions were used to switch between the movement and rest PLS regression models and the actual speed predictions were used to modulate the predicted velocity vectors. Therefore, any statistical differences between the actual and surrogate predictions were due to the ability of the PLS model to predict the time course and direction of movements and not from the ability of the logistic regression model to classify movement and rest.

##### Activation patterns.

Because the *z*-score calculation equalized the variance of each feature used within the prediction models, the model weights could be used to evaluate the importance of each feature type and cortical location. Because the model weights are the optimal combination of features to decode kinematics, they do not necessarily represent the strength of the relationship between an ECoG feature and a kinematic variable. To account for this, activation patterns describing the encoding of kinematic parameters by each ECoG feature were calculated as follows (Haufe et al., 2014):

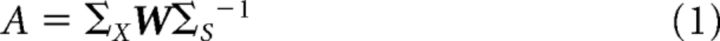
 Where **W** and **A** are matrices of model weights and activation patterns, respectively, Σ_X_ is the ECoG covariance matrix, and Σ_S_ is the kinematic data covariance matrix. Activation patterns were calculated for the both the logistic regression model predicting movement from rest and the movement period PLS model predicting speed and velocity for each of the 100 training sets. Because the knee of the skree plot representing the absolute activation pattern weight for each patient and kinematic parameter occurred at a threshold of 15–25% of the individual features, the 25% of features with the largest amplitude weights in each patient were used to calculate the relative importance of channels and features.

The relative importance of each channel was calculated by dividing the sum of the absolute value of activation patterns for a particular channel by the global sum of activation patterns in the top 25% of activation pattern magnitudes as follows:

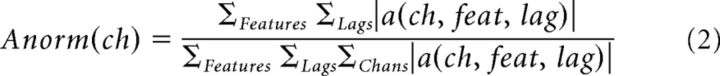
 The normalized activation patterns for each channel across each of the 100 training sets were then normalized between 0 and 1 and averaged across each of the 100 training sets. Activation patterns from each patient were mapped onto a single atlas brain using a weighted spherical Gaussian kernel centered at each electrode location. Areas with overlapping coverage across patients were combined using a weighted average based upon the distance from each electrode. Activation patterns for the logistic regression model classifying movement and rest, the PLS model predicting movement speed, and the PLS model predicting movement velocity were plotted separately with the activation pattern weights for the individual components of velocity averaged onto a single atlas brain.

To evaluate the importance of each ECoG feature type, the relative importance of each feature type was calculated by dividing the sum of the absolute value of activation pattern weights for a particular feature by the global sum of activation patterns in the top 25% of activation pattern magnitudes as follows:

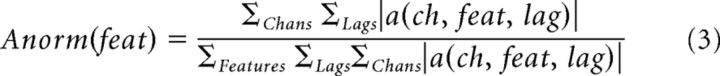
 The normalized activation patterns for each feature were then normalized between 0 and 1 and distributions of the normalized activation pattern weights across cross folds and patients were plotted for each feature for the logistic regression model, the PLS regression for speed, and the PLS regression for the velocity components. Finally, to evaluate the importance of each time lag, for all channels and frequencies with activation patterns in the top 25% of activation pattern magnitudes, the lag with the peak activation pattern magnitude was calculated and the proportion of peak lags at each lag tested was plotted. The importance of each location, feature type, and lag was evaluated separately for the two hands to compare the encoding of contralateral and ipsilateral arm movement kinematics. The similarity between the activation patterns associated with contralateral and ipsilateral movements was quantified by calculating the absolute value of the average activation patterns across the 100 training sets and calculating the correlation coefficient (Pearson's *r*) between the contralateral and ipsilateral arm conditions in each patient.

### Cross-prediction

Because we observed very similar activation patterns associated with contralateral and ipsilateral movement predictions, we used a cross-prediction method to determine whether information was conserved between the contralateral and ipsilateral arm movements. For each cross-prediction, the model trained using each contralateral arm movement training set was tested using the neural activity and kinematics from an ipsilateral arm test set and vice versa. For the cross-prediction, all time lags used in training the model were maintained when testing on the opposite arm. Cross-prediction accuracies were calculated as the correlation coefficient between the actual and predicted speed and velocity, as well as the percentage of predicted trajectories ending in the correct quadrant. As before, 100 temporal surrogate models were generated and 100 randomly shuffled channel and frequency assignments for each cross fold were used to evaluate whether the cross-prediction accuracies were significantly better than chance. As described previously, Bonferroni correction was used to correct for the total number of predicted kinematic variables (speed, Vx, Vy, Vz, and percentage targets hit) the number of regular hand predictions, the number of cross-prediction conditions, and the number of surrogate methods tested for the whole group and each individual patient (215 comparisons).

### Data and code availability

All data supporting the findings of this study and custom C++ and MATLAB (RRID:SCR_001622) code used to collect and analyze the data will be made available from the corresponding authors upon reasonable request.

## Results

### Behavioral performance

Patient participation was determined by their stamina and clinical needs, resulting in 104–288 trials with each hand (Table 1). Despite differences in the number of trials, all patients were able to consistently and accurately perform reaching movements to the target locations with similar movements for each arm. After excluding trials with reaction times >2 SDs from the mean, median reaction times for contralateral and ipsilateral arms differed by <100 ms in each patient, indicating that predictions were not driven by differences in attention or reaction speed between arms. Additionally, the median peak movement speed for the contralateral and ipsilateral arms differed by at most 3 cm/s in each individual patient, indicating that predictions were not affected by differences in movement speed between the contralateral and ipsilateral arm. To verify that the task required movements throughout the 3D workspace, we concatenated the kinematic data across trials and calculated the principle components of the seven component kinematic parameters considered: speed, velocity (V_x_, V_y_, and V_z_), and position (X, Y, and Z). The first four principle components explained an average of 24%, 22%, 18%, and 13.5% for the contralateral arm and 24.75%, 21.25%, 19.25%, and 13% for the ipsilateral arm, indicating that the eight targets used involved movements in multiple independent directions that were not systematically correlated with each other or movement speed.

### Movement-related cortical activity

**Figure 2.**
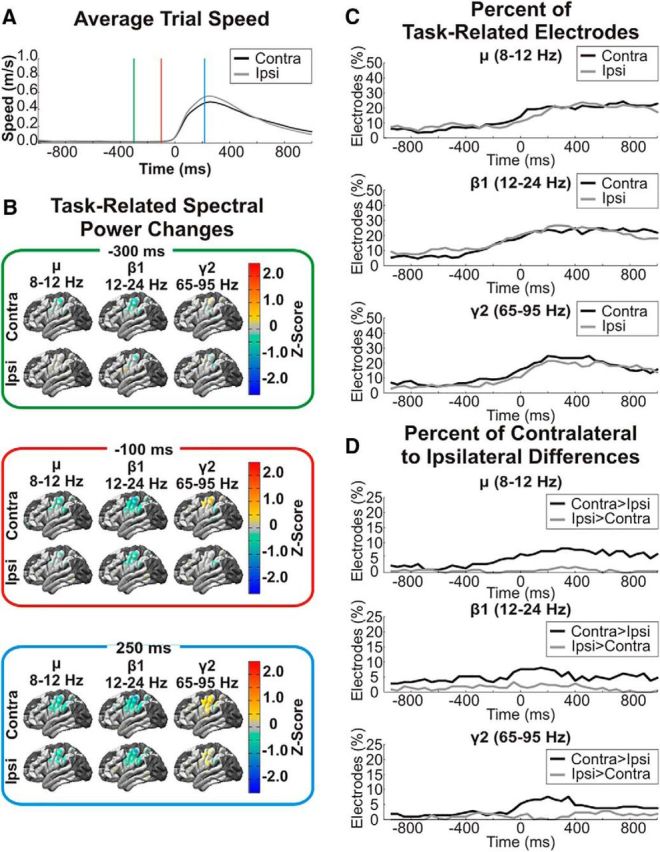
Movement-related spectral power changes. ***A***, After aligning to the movement onset, movement speed was averaged across trials and patients for the contralateral and ipsilateral hand showing similar amplitudes and time courses of reaching movements. The vertical lines indicate time windows for topographical plots of spectral power changes shown in ***B***. ***B***, Trial-averaged *z*-scores of log-transformed mu, beta, and high-gamma power changes that are significantly (*p* < 0.05) different from baseline are plotted for all electrodes at time windows 300 ms before movement onset (top), 100 ms before movement onset (middle), and 250 ms after movement onset (bottom). Movement-related decreases in mu and beta band power and movement-related increases in high-gamma band power are observed over sensorimotor cortex for both contralateral and ipsilateral reaches but begin earlier and are greater in amplitude for contralateral arm reaches. ***C***, The difference in the timing of movement-related spectral power changes was quantified by calculating the percentage of electrodes with *z*-scores significantly (*p* < 0.05) different from 0 at each time window. Additionally, correlations between the time courses of the percentage of active electrodes during contralateral and ipsilateral arm movements were calculated at various time lags to determine the time lag with the peak correlation. Correlations >0.9 were observed for the mu, beta 1, and gamma 2 frequencies with peak time lags of 50 ms (contralateral leading), −100 ms (ipsilateral leading), and 50 ms (contralateral leading). Therefore, whereas there were slight differences in the timing of gross movement-related neural activity, these time differences were not consistent across frequencies. ***D***, The difference in amplitude of movement-related spectral power changes was quantified by calculating the percentage of electrodes with significantly different spectral power changes between contralateral and ipsilateral arm movements. In mu, beta, and high-gamma bands, a subset of active electrodes demonstrate significantly (*p* < 0.05) greater amplitude spectral power changes during contralateral arm movements relative to ipsilateral arm movements.

Each patient had electrodes that demonstrated significant (*p* < 0.05) movement-related changes in cortical activity, particularly for the LMP, mu, beta, and high-gamma (gamma 2 and gamma 3) features. Specifically, the location of movement-related activity was centered in the primary motor cortex for both arms (Fig. 2*B*). Although the time and amplitude of movement speed was similar for each arm, the time courses of power changes were slightly different for the contralateral and ipsilateral arms (Fig. 2*C*), but these differences in the time course of neural activity were not consistent across frequencies. For mu, beta 1, gamma 2, and LMP features, correlation coefficients >0.9 were observed between lagged time courses of the percentage of active electrodes during contralateral and ipsilateral arm movements. Specifically, the peak correlation and time lag for these frequencies were as follows: mu: *r* = 0.94, lag = 50 ms (contralateral leading); beta 1: *r* = 0.91, lag = −100 ms (ipsilateral leading); gamma 2: *r* = 0.94, lag = 50 ms (contralateral leading); LMP: *r* = 0.92, lag = 0 ms. Therefore, whereas the time courses of spectral power changes were correlated for contralateral and ipsilateral limb movements, the small timing differences between these power changes were not consistent across features. In contrast, when comparing the magnitude of the gross neural correlates of contralateral and ipsilateral arm movements, whereas 20–30% of electrodes demonstrated significant movement-related changes in cortical activity for both limb movements, for a subset of electrodes (5–10%), the amplitude of movement-related changes was greater during contralateral arm reaches than during ipsilateral reaches (Fig. 2*D*).

### Prediction of ipsilateral movement kinematics

**Figure 3.**
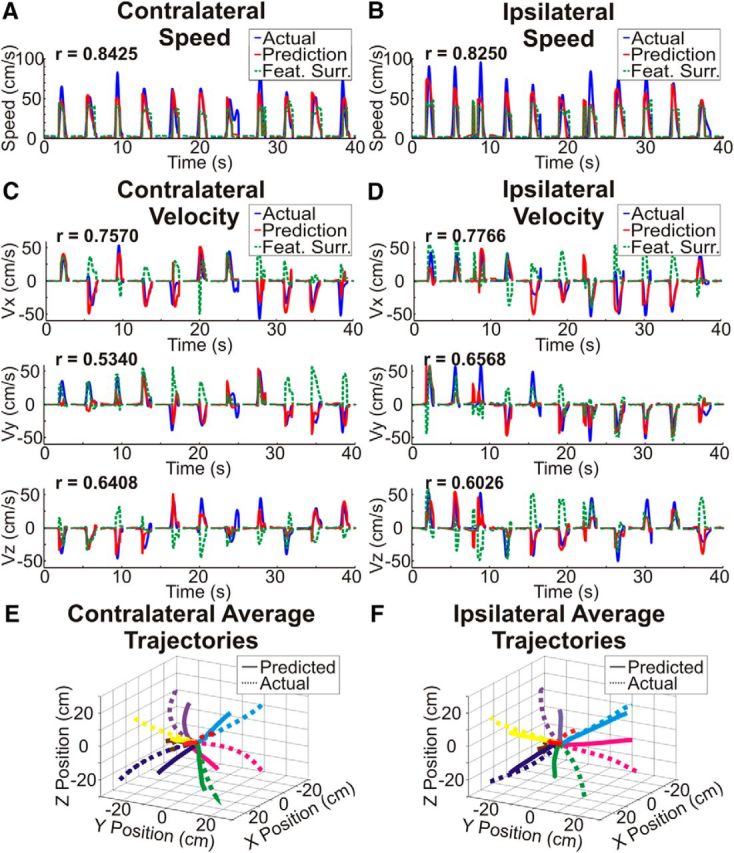
Exemplar kinematic predictions. ***A***–***D***, Exemplar kinematic predictions of contralateral arm movement speed (***A***), ipsilateral arm movement speed (***B***), contralateral arm movement velocity (***C***), and ipsilateral arm movement velocity (***D***). Actual kinematic traces are shown in blue, predicted kinematic traces are shown in red, and an example surrogate prediction with reshuffled feature weights is shown in green. The plots were generated using consecutive trials from a single contralateral and ipsilateral test set from Patient 4. Kinematic predictions were made from 2 s before movement onset to the end of each trial and show accurate predictions of 3D kinematics for both the contralateral and ipsilateral arm. ***E***, ***F***, Movement trajectories were generated by concatenating predicted velocities within each trial, normalizing the trajectory times across trials, and averaging all trajectories for each target location. Averaged trajectories were generated using every test set from Patient 4 and end in the correct quadrant for each target for both contralateral and ipsilateral arm movements.

Next, we tested whether the cortical activity observed during ipsilateral arm reaches could be extended beyond gross movement-related activity and used to predict specific kinematics (speed, velocity, and position) of ipsilateral arm reaches on a time-point by time-point basis and examined how the ECoG prediction of ipsilateral arm reaches differed from the prediction of contralateral movement kinematics. Figure 3 shows exemplar contralateral and ipsilateral movement predictions for the full model prediction and a surrogate model prediction generated by reshuffling the feature weights. Predictions were made for consecutive trials from a single test fold from Patient 4. The figure demonstrates that 3D movement kinematics for both contralateral and ipsilateral reaches could be predicted with very high accuracies. Because the actual logistic regression predictions were used to switch between the movement and rest PLS regression models and the actual predicted speed was used to modulate velocity for both actual and surrogate predictions, both the actual and surrogate predictions predict the movement and rest periods well. Therefore, the differences between actual and surrogate kinematic predictions observed were driven by the ability to predict the actual time courses of speed and velocity and not by the ability to predict movement and rest periods. The predicted trajectories shown in Figure 3, *E* and *F*, were made by averaging the predicted trajectories from all trials to each target respectively. Because the magnitude of the predicted velocity vector was set to the predicted movement speed, which often undershot the actual movement speed, the predicted trajectories are often shorter than the actual trajectories. This low predicted speed was likely caused by the combination of relatively long temporal windows for spectral estimation that may not have captured sharp changes in high-frequency power, training the model to predict the entire time course of movement speed instead of only the peak movement speed, and the use of a linear model that allowed us to easily interpret the model weights, but may not have perfectly fit the relationship between neural activity and movement kinematics. Additionally, both correct and incorrect predictions were averaged together, which could cause additional decreases in the average predicted trajectory lengths relative to the actual average trajectory lengths. To evaluate the relative contribution of these two factors, we compared the length of the average predicted and actual trajectories (Fig. 3*E*,*F*) with the average length of the predicted trajectories from each individual trial. From this analysis, we found that 25–30% of the difference between the lengths of the average trajectories could be accounted for by the average length of the individual predicted trajectories being shorter than the actual trajectories. This indicates that both factors contributed to the average predicted trajectories being shorter than the average actual trajectories. Despite the difference in length of the average actual and predicted trajectories, as the average trajectory for each target is clearly in the same quadrant as the actual averaged trajectory it is clear that, on average, the movement directions are predicted accurately.

**Figure 4.**
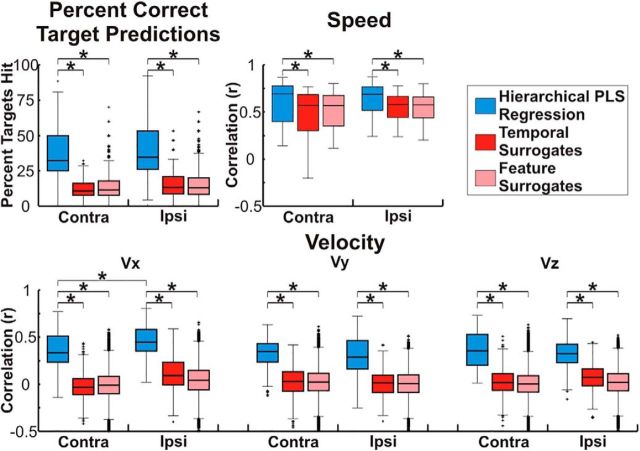
Prediction accuracy. Prediction accuracy was assessed by calculating the percentage of trajectories ending in the correct quadrant as well as calculating the correlation coefficient (Pearson's *r*) between the observed and predicted kinematics (speed, Vx, Vy, and Vz). Prediction accuracies were calculated for each of the 100 random test sets for each patient and chance accuracy was determined using two different surrogate datasets. Accuracies were combined across patients and randomly selected test sets. Boxes show the median, 25^th^ percentile, and 75^th^ percentile of accuracy. Whiskers show the range of accuracies with outliers >2.7 SDs indicated by a “+” symbol. Comparisons that are statistically significant (*p* < 0.05) after Bonferonni correction for the total number of comparisons are indicated by a “*” symbol. Across patients, the prediction accuracy and correlations between predicted and actual speed, Vx, Vy, and Vz were all significantly (*p* < 0.05) better than both surrogate distributions even after Bonferroni correction for the total number of true and cross-prediction comparisons. Furthermore, in addition to the comparisons across patients shown here, the prediction accuracies and correlations between the predicted and actually kinematics were also significantly better than surrogate predictions for each individual patient (see extended data Fig. 4-1, and Fig. 4-2).

Across patients, predicted movement kinematics (speed and velocity) were significantly (*p* < 0.05) more correlated with the actual kinematics and the percentage of targets predicted was significantly better (*p* < 0.05) than two surrogate predictions, even after Bonferroni correcting for the total number of comparisons. Distributions of full model and surrogate prediction accuracies are shown in Figure 4 and statistical tests comparing these accuracies are summarized in Table 2. Additionally, distributions and statistical tests comparing full model and surrogate prediction accuracies for individual patients are included in the extended data (Fig. 4-1, and Fig. 4-2). Although the prediction accuracies were impacted by the unique electrode coverage, movement predictions were significantly better than chance for each individual patient. For speed, because both original model and surrogate predictions used the original model logistic regression to switch between the movement and rest models, both the full model and surrogate predictions have correlations with the actual movement speed that are >0. Despite this, the full model prediction fit the time course of speed better than the surrogate prediction, leading to significantly higher correlation coefficients between predicted and actual speed for the full model when compared with the surrogate predictions. For velocity, because the eight targets incorporated movements in both directions along all three axes, the correlations between full model predictions and the actual velocity were >0, whereas correlations between the surrogate model predictions and velocity were centered ∼0. For the percentage of targets predicted in our eight target center-out task, chance prediction was 12.5%. This chance level was confirmed by the two surrogate methods that both clustered ∼12.5% targets predicted correctly for both the contralateral and ipsilateral arm. Although lower in amplitude than the differences between the actual and surrogate predictions the anterior–posterior component of velocity was significantly more accurately predicted for ipsilateral limb movements than for contralateral limb movements. The prediction accuracies for all other kinematic parameters were not significantly different between the contralateral and ipsilateral limbs after multiple comparison correction [speed (W(400,400) = 156073, *z* = −1.26, *p* = 0.2067), Vx (W(400,400) = 132691, *z* = −8.42, *p* = 3.8 × 10^−17^), Vy (W(400,400) = 167894, *z* = 2.35, *p* = 0.0186), Vz (W(400,400) = 167989, *z* = 2.38, *p* = 0.0172), targets hit (W(400,400) = 157019.5, *z* = −0.97, *p* = 0.3303)].

10.1523/JNEUROSCI.0015-18.2018.f4-1 Figure 4-1 **Individual patient prediction accuracy.** Prediction accuracy for each individual patient was assessed by calculating the percent of trajectories ending in the correct quadrant as well as calculating the correlation coefficient (Pearson’s r) between the observed and predicted kinematics (speed, Vx, Vy, and Vz). Prediction accuracies were calculated for each of the 100 random test sets for each patient and chance accuracy was determined using 2 different surrogate datasets. Boxes show the median, 25^th^ percentile, and 75^th^ percentile of accuracy. Whiskers show the range of accuracies with outliers greater than 2.7 standard deviations indicated by a + symbol. With the exception of speed for patient 1, the prediction accuracy and correlations between predicted and actual speed, Vx, Vy, and Vz were significantly (p<0.05) better than both surrogate distributions for each patient, even after Bonferonni correction for the total number of true and cross-prediction comparisons across all patients. Download Figure 4-1, TIF file

10.1523/JNEUROSCI.0015-18.2018.f4-2 Figure 4-2 **Individual patient prediction statistics.** Full model predictions and surrogate predictions were compared for each individual patient using a rank-sum test. Median prediction accuracies, Wilcoxon rank-sum statistics (W), z-statistics, and p-values for each comparison are shown. Statistically significant differences after Bonferonni correcting for the total number of comparisons tested are highlighted in bold. Download Figure 4-2, DOCX file

**Table 2.** Full model prediction statistics

	Actual	Temporal surrogates			Feature surrogates		
	Median	Median	Effect size	*p*	Median	Effect size	*p*
**Contralateral**							
Speed (Pearson's *r*)	0.6922	0.571	**W(400,400) = 191459, *z* = 9.56**	**1.1×10**^−21^	0.5687	**W(400,40000) = *10767018*,z = 11.58**	**5.4×10**^−31^
Vx (Pearson's *r*)	0.3429	−0.0257	**W(400,400) = 232199, *z* = 22.03**	**1.4×10**^−107^	−0.0039	**W(400,40000) = 15293940, *z* = 31.08**	**4.4×10**^−212^
Vy (Pearson's *r*)	0.3523	0.0324	**W(400,400) = 227553, *z* = 20.61**	**2.3×10**^−94^	0.0271	**W(400,40000) = 14925373, *z* = 29.49**	**3.5×10**^−191^
Vz (Pearson's *r*)	0.3609	0.0216	**W(400,400) = 228663, *z* = 20.95**	**1.9×10**^−97^	0.007	**W(400,40000) = 15135199, *z* = 30.40**	**6.0×10**^−203^
Targets hit (%)	32.26%	10.71%	**W(400,400) = 228749, *z* = 20.99**	**8.6×10**^−98^	11.54%	**W(400,40000) = 14792910, *z* = 28.96**	**1.8×10**^−184^
**Ipsilateral**							
Speed (Pearson's *r*)	0.6893	0.5774	**W(400,400) = 187830, *z* = 8.45**	**2.8×10**^−17^	0.5756	**W(400,40000) = 10928459, *z* = 12.27**	**1.3×10**^−34^
Vx (Pearson's *r*)	0.4553	0.0972	**W(400,400) = 230725, *z* = 21.58**	**2.8×10**^−103^	0.0477	**W(400,40000) = 15707188, *z* = 32.86**	**7.8×10**^−237^
Vy (Pearson's *r*)	0.2942	0.0184	**W(400,400) = 223823, *z* = 19.47**	**2.0×10**^−84^	0.0115	**W(400,40000) = 14458468, *z* = 27.48**	**2.9×10**^−166^
Vz (Pearson's *r*)	0.3281	0.0767	**W(400,400) = 225494, *z* = 19.98**	**8.3×10**^−89^	0.0233	**W(400,40000) = 15126672, *z* = 30.36**	**1.8×10**^−202^
Targets hit (%)	34.78%	13.33%	**W(400,400) = 219293, *z* = 18.09**	**3.6×10**^−73^	13.04%	**W(400,40000) = 14316072.5, *z* = 26.90**	**2.4×10**^−159^

Full model predictions and surrogate predictions were compared using a rank–sum test. Median prediction accuracies, Wilcoxon rank–sum statistics (W), *z*-statistics, and *p*-values for each comparison are shown. All comparisons were statistically significant after Bonferroni correcting for the total number of comparisons tested.

**Figure 5.**
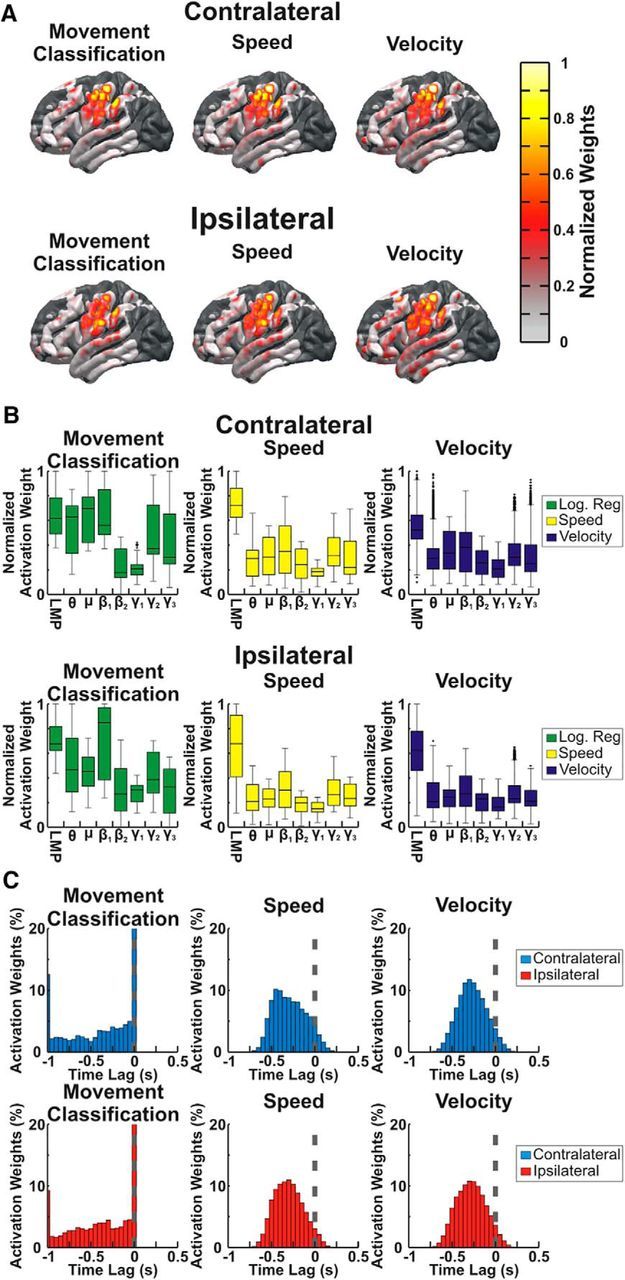
Spatial, spectral, and temporal importance. The importance of individual locations, frequencies, and time lags for predicting kinematics was determined by converting prediction model weights to activation patterns for the logistic regression classifying movement and rest (movement classification) and movement-period PLS regression. ***A***, Normalized activation pattern weights for the top 25% of weights are plotted on an atlas brain using a Gaussian kernel centered at each electrode site. Activation patterns across patients were combined onto a single atlas brain and areas with overlapping coverage were combined across patients using a weighted average based upon the distance from each electrode. Additionally, the normalized activation pattern weights were averaged across velocity components to produce plots for velocity. For each kinematic component, the most important cortical locations are centered over the central sulcus in primary sensorimotor cortex for both hands. ***B***, Normalized activation pattern weights for the top 25% of weights across all electrodes, patients, and cross folds were combined for each feature type. Distributions of activation pattern weights are plotted with boxes showing the median, 25^th^ percentile, and 75^th^ percentile and whiskers showing the extent of weights. Outliers >2.7 SDs from the median are shown with a “+” symbol. Low frequencies including the beta band and LMP had the largest normalized activation patterns for movement classification, whereas movement kinematics (speed and velocity) were represented most strongly within LMP features followed by beta and high-gamma band features for both contralateral and ipsilateral arm movements. ***C***, For channels and frequencies with activation pattern weights in the top 25% of weights, the time lag between neural activity and kinematics with the peak activation weight magnitude was determined. Histograms show the proportion of weights at each time lag. The logistic regression weights had a peak at a time lag of 0 s. For speed and velocity, the neural activity led the kinematics that were predicted with the majority of time lags falling between −500 ms and 0 s for both contralateral and ipsilateral arm movements.

As shown in Figure 5, activation patterns for movement classification, speed, and velocity were strongest in cortical areas centered over the primary motor cortex. Additionally, whereas movement classification was encoded most within low frequencies (LMP, mu, and beta), for speed and velocity decoding, the strongest activation pattern weights were observed for the LMP, followed by both low (beta and mu) and high (gamma 2 and gamma 3) frequency spectral power changes. Furthermore, whereas movement classification was encoded most at a time lag of 0 ms, speed and velocity were encoded predominantly at time lags between −500 and 0 ms with the neural activity leading the encoded kinematic parameters. Contrary to our initial hypothesis, the spatial, spectral, and temporal encoding of kinematics was similar for contralateral and ipsilateral arm movements. Specifically, the average correlation coefficient relating the absolute value of activation pattern weights for the contralateral and ipsilateral arm movements was 0.57 for the logistic regression model classifying movement and rest, 0.49 for the PLS model predicting speed, and 0.36, 0.32, and 0.35 for the PLS models predicting Vx, Vy, and Vz respectively. Correlation coefficients are summarized in Table 3 and were significantly different from 0 for each kinematic component and patient, showing that similar features and time lags were important for predicting movement kinematics of both contralateral and ipsilateral arm movements.

**Table 3.** Activation pattern weight correlations

Patient	Logistic Regression		Speed		Vx		Vy		Vz	
	*r*	*p*	*r*	*p*	*r*	*p*	*r*	*p*	*r*	*p*
1	0.4	**5.2** × **10**^−9^	0.6	**<1.0** × **10**^−324^	0.55	**<1.0** × **10**^−324^	0.52	**<1.0** × **10**^−324^	0.31	**1.5** × **10**^−140^
2	0.46	**1.1** × **10**^−30^	0.19	**2.3** × **10**^−132^	0.16	**5.3** × **10**^−98^	0.13	**3.6** × **10**^−64^	0.16	**4.5** × **10**^−94^
3	0.62	**1.7** × **10**^−51^	0.46	**<1.0** × **10**^−324^	0.25	**1.4** × **10**^−204^	0.26	**3.0** × **10**^−216^	0.26	**3.4** × **10**^−215^
4	0.81	**3.6** × **10**^−124^	0.7	**<1.0** × **10**^−324^	0.5	**<1.0** × **10**^−324^	0.37	**<1.0** × **10**^−324^	0.62	**<1.0** × **10**^−324^

Correlation coefficients (Pearson's *r*) were calculated between the absolute value of activation pattern weights for contralateral and ipsilateral arm prediction models for each patient and kinematic parameter, showing good correspondence between the contralateral and ipsilateral prediction models. All comparisons were statistically significant after Bonferroni correcting for the total number of comparisons tested.

### Cross-prediction

**Figure 6.**
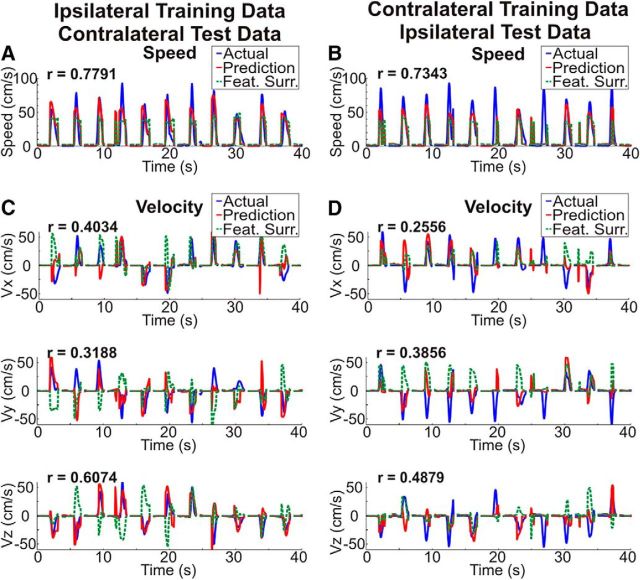
Exemplar cross-prediction accuracy. ***A***–***D***, Exemplar kinematic cross-predictions were generated by using ipsilateral reaching movements to train our model and predict contralateral movement speed (***A***) and velocity (***C***) and using contralateral movements to train a model to predict ipsilateral arm speed (***B***) and velocity (***D***). Actual kinematic traces are shown in blue, predicted kinematics traces are shown in red, and an example surrogate prediction with reshuffled feature weights is shown in green. The plots were generated using consecutive test set trials from a single test set from Patient 4. Kinematics predictions were made from 2 s before movement onset to the end of each trial and show accurate predictions of 3D kinematics even when the prediction model was trained using reaching movements from the opposite hand.

**Figure 7.**
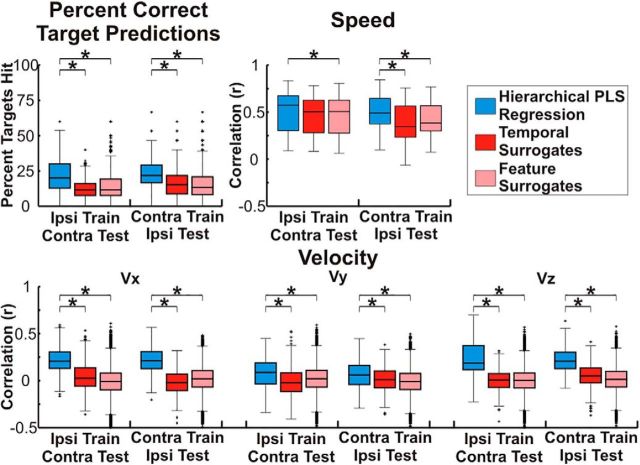
Cross-prediction accuracy. Cross-prediction accuracy was assessed by calculating the percentage of trajectories ending in the correct quadrant as well as calculating the correlation coefficient (Pearson's *r*) between the observed and predicted kinematics (speed, Vx, Vy, and Vz). Accuracies were combined across patients and random test sets and the boxes show the median, 25^th^ percentile, and 75^th^ percentile of accuracy. Whiskers show the range of accuracies with outliers >2.7 SDs indicated by a “+” symbol. Comparisons that are statistically significant (*p* < 0.05) after Bonferonni correction for the total number of comparisons are indicated by a “*” symbol. Across patients, with the exception of the comparison between the 'ipsilateral training, contralateral testing' prediction and the temporal surrogate prediction for speed, the cross-prediction accuracy and correlations between predicted and actual speed, Vx, Vy, and Vz were all significantly (*p* < 0.05) better than either surrogate even after Bonferroni correction for the total number of comparisons, showing that some components of the ECoG representation of kinematics are conserved within a single cortical hemisphere for contralateral and ipsilateral arm movements.

**Figure 8.**
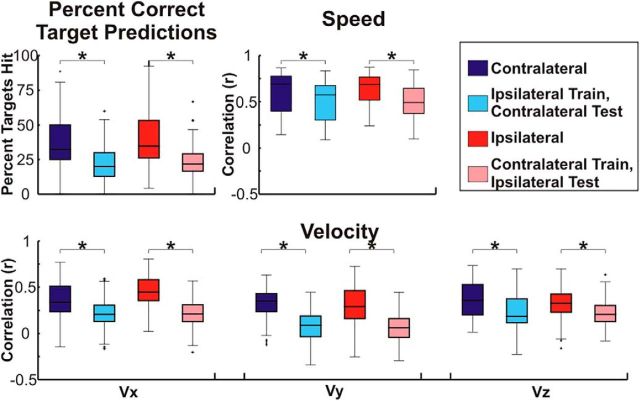
Comparison of true and cross-prediction accuracies. True and cross-prediction accuracy were assessed by calculating the percentage of trajectories ending in the correct quadrant as well as calculating the correlation coefficient (Pearson's *r*) between the observed and predicted kinematics (speed, Vx, Vy, and Vz). Accuracies were combined across patients and random test sets and the boxes show the median, 25^th^ percentile, and 75^th^ percentile of accuracy. Whiskers show the range of accuracies with outliers >2.7 SDs indicated by a “+” symbol. Comparisons that are statistically significant (*p* < 0.05) after Bonferonni correction for the total number of comparisons are indicated by a “*” symbol. Across patients, whereas the accuracy and correlations between predicted and actual speed, Vx, Vy, and Vz were all significantly (*p* < 0.05) better than chance for both true and cross-predictions, true predictions were significantly (*p* < 0.05) better than the respective cross-predictions even after Bonferroni correction for the total number of comparisons.

Because of the similarity in the cortical locations and features showing the strongest activation pattern weights, we used a cross-prediction analysis to determine whether the encoding of kinematic information is conserved between arms within a single hemisphere. Exemplar cross-predictions and surrogate cross-predictions are shown in Figure 6 for Patient 4. Although poorer in accuracy than the original predictions (Fig. 3), it is clear that both cross-predictions isolate movement from rest and accurately predict the speed and velocity of movements. Across patients, with the exception of the temporal surrogate series for speed, the percentage of cross-prediction trajectories ending in the correct quadrant and the correlations between the actual speed and velocity and the cross-predictions were all significantly better than chance (*p* < 0.05) even after Bonferroni correcting for the total number of comparisons across all 4 true and cross-prediction conditions. Distributions of actual and surrogate prediction accuracies are shown in Figure 7 and statistical tests comparing these accuracies are summarized in Table 4. Although the cross-prediction accuracies were significantly better than chance, as shown in Figure 8 and summarized in Table 5, unsurprisingly, the true predictions were significantly (*p* < 0.05) more accurate than the cross-predictions for the percentage of targets hit and each of the kinematic parameters tested.

**Table 4.** Cross-prediction statistics

	Actual	Temporal surrogates			Feature surrogates		
	Median	Median	Effect Size	*p*	Median	*W*	*p*
**Ipsilateral training, contralateral test**							
Speed (Pearson's *r*)	0.5749	0.5056	W(400,400) = 171489, *z*=3.45	5.5×10^−4^	0.508	**W(400,40000) = 9327699, *z* = 5.38**	**7.7×10**^−8^
Vx (Pearson's *r*)	0.2128	0.0307	**W(400,400) = 208614, *z* = 14.81**	**1.2×10**^−49^	−0.0043	**W(400,40000) = 14088014, *z* = 25.89**	**9.8×10**^−148^
Vy (Pearson's *r*)	0.0887	−0.0224	**W(400,400) = 187350, *z* = 8.31**	**9.8×10**^−17^	0.0218	**W(400,40000) = 9970769, *z* = 8.15**	**3.8×10**^−16^
Vz (Pearson's *r*)	0.1924	0.012	**W(400,400) = 220591, *z* = 18.48**	**3.0×10**^−76^	0.0074	**W(400,40000) = 14031911, *z* = 25.64**	**5.0×10**^−145^
Targets Hit (%)	20.00%	11.54%	**W(400,400) = 198405, *z* = 11.70**	**1.2×10**^−31^	11.54%	**W(400,40000) = 11539797, *z* = 14.93**	**2.1×10**^−50^

	Actual	Temporal surrogates			Feature surrogates		
	Median	Median	*W*	*p*	Median	*W*	*p*
**Contralateral training, ipsilateral test**							
Speed (Pearson's *r*)	0.4929	0.3487	**W(400,400) = 188745, *z* = 8.73**	**2.4×10**^−18^	0.3851	**W(400,40000) = 10355356, *z* = 9.80**	**1.1×10**^−22^
Vx (Pearson's *r*)	0.2146	−0.018	**W(400,400) = 223743, *z* = 19.44**	**3.3×10**^−84^	0.0225	**W(400,40000) = 13819858, *z* = 24.73**	**5.1×10**^−135^
Vy (Pearson's *r*)	0.062	0.0112	**W(400,400) = 175663, *z* = 4.73**	**2.2×10**^−6^	−0.0092	**W(400,40000) = 10329137, *z* = 9.69**	**3.3×10**^−22^
Vz (Pearson's *r*)	0.2074	0.0511	**W(400,400) = 211047, *z* = 15.56**	**1.4×10**^−54^	0.0149	**W(400,40000) = 14091735, *z* = 25.90**	**6.5×10**^−148^
Targets hit (%)	21.74%	15.38%	**W(400,400) = 191739, *z* = 9.66**	**4.5×10**^−22^	13.33%	**W(400,40000) = 11614574, *z* = 15.24**	**1.8×10**^−52^

Cross-predictions and surrogate cross-predictions were compared using a rank–sum test. Median prediction accuracies, Wilcoxon rank–sum statistics (W), *z*-statistics, and *p*-values for each comparison are shown. With the exception of the temporal surrogate predictions for speed in the 'ipsilateral training, contralateral testing' condition, all other comparisons between actual and surrogate cross-predictions were statistically significant even after Bonferroni correcting for the total number of comparisons tested.

**Table 5.** Comparison of true and cross-prediction results

	Contralateral				Ipsilateral			
	Actual	Cross-prediction	Effect size	*p*	Actual	Cross-prediction	Effect size	*p*
Speed (Pearson's *r*)	0.6922	0.5749	**W(400,400) = 187859, *z* = 8.46**	**2.6×10**^−17^	0.6893	0.4929	**W(400,400) = 189087, *z* = 8.84**	**9.6×10**^−19^
Vx (Pearson's *r*)	0.3429	0.2128	**W(400,400) = 199535, *z* = 12.04**	**2.3×10**^−33^	0.4553	0.2146	**W(400,400) = 222973, *z* = 19.21**	**3.2×10**^−82^
Vy (Pearson's *r*)	0.3523	0.0887	**W(400,400) = 223799, *z* = 19.46**	**2.4×10**^−84^	0.2942	0.062	**W(400,400) = 212251, *z* = 15.93**	**4.1×10**^−57^
Vz (Pearson's *r*)	0.3609	0.1924	**W(400,400) = 195735, *z* = 10.87**	**1.5×10**^−27^	0.3281	0.2074	**W(400,400) = 186848, *z* = 8.15**	**3.5×10**^−16^
Targets hit (%)	32.26%	20.00%	**W(400,400) = 199140, *z* = 11.92**	**9.4×10**^−33^	34.78%	21.74%	**W(400,400) = 204855, *z* = 13.67**	**1.6×10**^−42^

True and cross-predictions were compared using a Wilcoxon rank–sum test. Median prediction accuracies, Wilcoxon rank–sum statistics (W), *z*-statistics, and *p*-values for each comparison are shown. Statistically significant differences after Bonferroni correcting for the total number of comparisons tested are highlighted in bold.

## Discussion

This study provides the first demonstration that human ECoG signals can be used to decode 3D reaching kinematics of the ipsilateral arm. Although gross movement-related power changes were observed for contralateral and ipsilateral reaches, the specific relationship between unilateral ECoG activity and movement kinematics was similar for both arms. These findings demonstrate that movement kinematics are distributed bilaterally across cortical hemispheres.

Importantly, when neural activity was related to specific kinematics, 3D kinematics of ipsilateral arm reaches were decoded with similar levels of accuracy as contralateral reaches. Although the ability to decode ipsilateral limb kinematics does not establish a causal role of the ipsilateral hemisphere for movement execution, a representation of specific movement features, such as kinematics, is a necessary condition for the ipsilateral hemisphere to play a causal role in movement execution. Previous studies have also found ipsilateral motor activations during a strictly unimanual task (Buetefisch et al., 2014). Furthermore, along with more severe contralesional deficits, stroke survivors also have ipsilesional motor deficits following unilateral lesions (Baskett et al., 1996; Sunderland, 2000; Schaefer et al., 2007, 2009a,b, 2012). When combined with these previous studies, our finding that 3D kinematics are encoded ipsilaterally strengthens the argument that the ipsilateral hemisphere plays an active role in motor control.

Interestingly, the cortical representations of movement speed and velocity were conserved across arms. Although previous studies have shown that neural patterns encoding ipsilateral limb movements are similar to those encoding contralateral limb movements with regard to the somatotopic organization of gross body parts (Fujiwara et al., 2017), the somatotopic organization of fingers (Scherer et al., 2009; Liu et al., 2010; Diedrichsen et al., 2013, 2018), and movement direction (Haar et al., 2017), this is the first study to show that specific kinematics of arm movements are bihemispherically represented on a time point by time point basis. As the encoding of specific movement parameters (i.e., kinematics or kinetics) is a necessary condition for the ipsilateral hemisphere to play an active role in movement execution, this is an important advancement and distinction. It should be noted that patients performed reaching movements with a single arm at a time. As neural activity during bimanual movements is not simply a linear combination of neural activity during unimanual movements (Tanji et al., 1988; Donchin et al., 1998; Kermadi et al., 2000; Diedrichsen et al., 2013; Gallivan et al., 2013), it is uncertain whether distinct cortical representations of contralateral and ipsilateral movement kinematics would be observed during bimanual tasks. Similarly, the dominant hemisphere has been associated with controlling movement dynamics, including trajectory control, of both arms (Sainburg and Kalakanis, 2000; Schaefer et al., 2007, 2009a,b, 2012). Because all electrodes were in the dominant hemisphere, further work will be necessary to isolate any differences in the encoding of ipsilateral limb movements between the dominant and nondominant hemispheres. Finally, ipsilateral motor activity has been posited to preferentially relate to proximal muscle activity (Colebatch et al., 1991; Jankelowitz and Colebatch, 2002). Our results appear to confirm this because the cross-decoding accuracies were higher for the velocity components along the superior–inferior and anterior–posterior axes, which would likely rely on proximal musculature. Because the task did not control for the muscles used, these results must be interpreted within the context of the activity of the entire motor system.

This study relied on patients with electrode locations determined by clinical needs. Because the electrodes did not extensively cover more medial areas of the motor or premotor areas, the electrode coverage was suboptimal for decoding reaching. Because the most important areas for decoding were in the most medial areas of the motor cortex, the statistically significant decoding shown is likely a baseline for what could be achieved with better coverage. Despite this suboptimal coverage, we were likely able to decode kinematics because large-amplitude movements are associated with greater modulation of neural activity (Waldvogel et al., 1999) and full arm movements involve muscles covering a greater extent of the motor homunculus than movements constrained to a single joint (Penfield and Boldrey, 1937). Additionally, whereas the ability to decode kinematics and the cortical locations that kinematics were encoded in were similar across arms, it is possible that there could be differences in other motor areas. However, because fMRI studies have found similar encoding of nonkinematic movement parameters in whole-brain analyses (Diedrichsen et al., 2013; Haar et al., 2017), we would hypothesize that the cortical encoding of contralateral and ipsilateral kinematics would still be similar regardless of the motor areas covered.

The ability to use ECoG to decode kinematics of the same-sided hand also underscores the possibility for a stroke survivor to use signals from their unaffected hemisphere to control a brain–computer interface (BCI). BCI systems for stroke have received increasing attention in recent years (Soekadar et al., 2015). Although the majority of studies have focused on using control signals from perilesional cortex (contralateral to the impaired limb), because the ability to modulate perilesional cortical activity decreases in severely affected patients (Buch et al., 2012), the unaffected (ipsilateral) hemisphere may provide a better control signal in significantly impaired patients. Additionally, whereas the role of the unaffected hemisphere in motor recovery after stroke has been debated (Weiller et al., 1992; Johansen-Berg et al., 2002; Ward et al., 2003a,b; Tecchio et al., 2006), training with a BCI-controlled exoskeleton controlled from the unaffected hemisphere can lead to functional improvements in chronic stroke survivors (Bundy et al., 2017). The similarity between the neural representations of contralateral and ipsilateral limb movements, however, creates an additional dimension for considering more advanced neuroprosthetic strategies. For rehabilitation, the similarity in ipsilateral and contralateral signals may be beneficial. Given that movement kinematics are represented in both hemispheres, there should theoretically still be parts of the brain that are present after a hemispheric insult that can be engaged to drive functional recovery. However, when considering an implant that is intended to perform more complex brain-derived control, the similarity in physiology could cause potential “cross-talk” with contralateral movement intentions. This interference could hamper a stand-alone BCI that replaces lost function (i.e., controls the paralyzed limb) rather than a BCI that simply facilitates endogenous plasticity mechanisms (i.e., recovery of natural limb). That said, future studies could test whether it is possible to separate ipsilateral and contralateral movement intentions with improved anatomic resolution or additional levels of hierarchy. Similarly, whereas this demonstration of the offline decoding of kinematics establishes the potential feasibility of BCIs, studies of online BCI control will be required to demonstrate that the algorithms and features used allow patients to actively adapt their neural activity to achieve online BCI control (Cunningham et al., 2011).

Although the findings of this study are exciting, there are several additional considerations to note. First, all of the patients had chronic epilepsy. However, care was taken to ensure that recordings were at least 2 h from any seizures. Furthermore, trials with interictal activity were removed before analysis and three of the four patients had epileptic foci located in the temporal lobe separate from the most important areas for decoding. Therefore, we believe that the results were not significantly affected by focal epileptic activity. Electrophysiological correlates of movements are also affected by a number of factors that may have been involved in this study. First, increased task complexity and effort increase movement-related cortical activations (Manganotti et al., 1998; Slobounov et al., 2004). Although reaction time and movement speed were similar between the two arms, all ECoG electrodes were located contralateral to the dominant hand. Therefore, the strong ipsilateral signals may have been related to increased effort for nondominant hand movements. Additionally, all patients performed the contralateral task before the ipsilateral task. However, it is unlikely that this would have affected the results because the number of trials was likely not large enough to result in significant training affects and the movements involved were natural reaching movements. Postural movements of the hemibody contralateral to the moving arm also represent a potential confound. However, because postural movements are unlikely to be conserved between right and left arm movements, the statistically significant cross-prediction of velocity across each dimension provides evidence that the observed results were not caused by contralateral postural movements. Furthermore, it is unlikely that postural stabilizing movements can fully account for the multiple degrees of freedom that were decoded. Finally, because of the interleaved rest and movement periods, whereas the results demonstrate the ability to predict ipsilateral movement kinematics with accuracies above chance, the prediction accuracies are affected by the ability to predict 3D movement kinematics and the ability to predict movement from rest and not the ability to predict movement kinematics in isolation.

Collectively, this study demonstrates that 3D kinematics of ipsilateral arm movements are encoded in human ECoG signals. Furthermore, the cortical representation of unilateral movement kinematics is conserved across arms. Therefore, these results strengthen evidence that the ipsilateral hemisphere plays a role in planning and executing voluntary motor movements with important implications for neuroprosthetic and neurorehabilitation applications.
