# Supplementary figures and images for: Unilateral, 3D Arm Movement Kinematics Are Encoded in Ipsilateral Human Cortex
Source: J Neurosci. 2018 Nov 21;38(47):10042–56. doi: 10.1523/JNEUROSCI.0015-18.2018 (PMC6246886; doi:10.1523/JNEUROSCI.0015-18.2018)

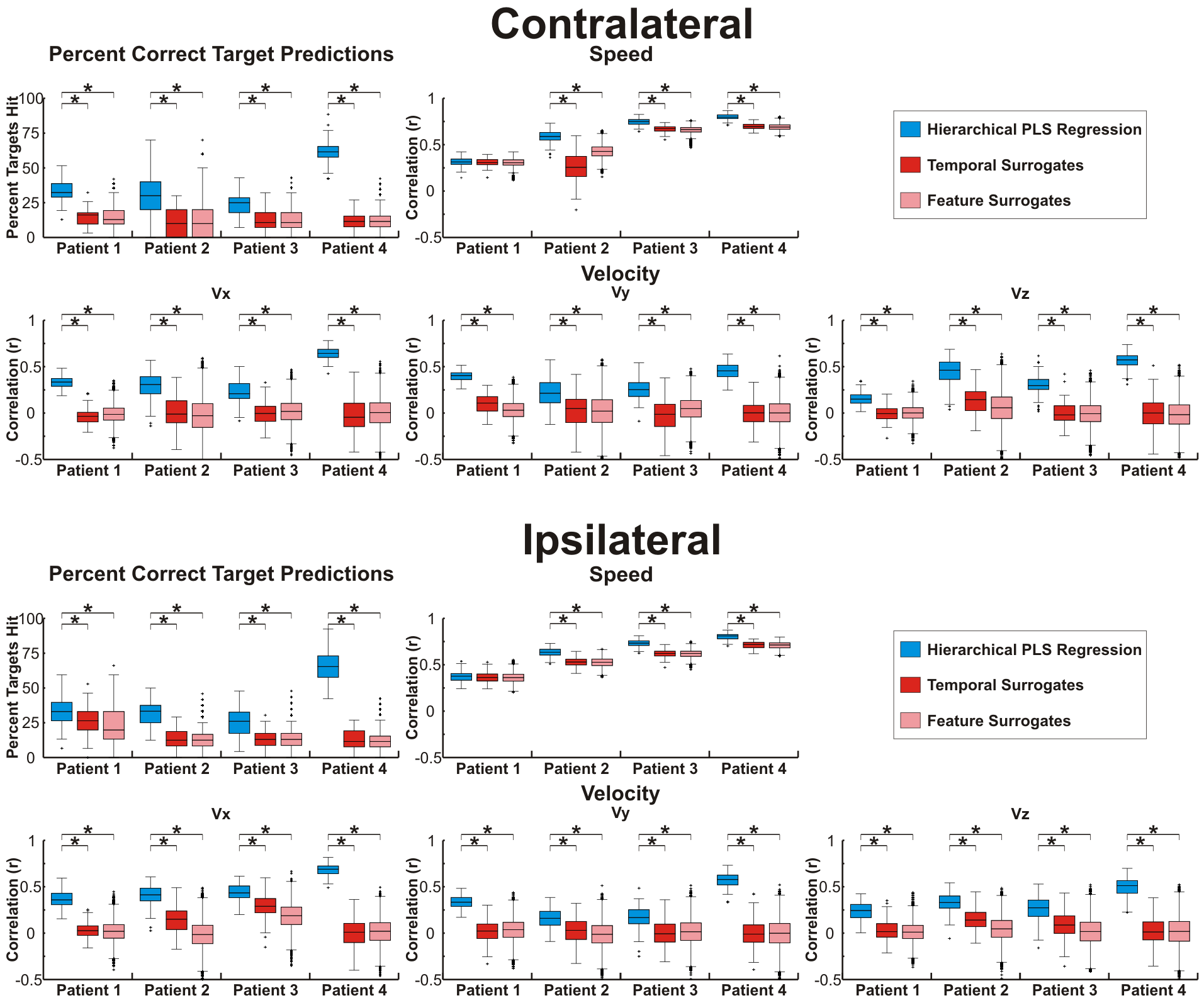

Supplement: Figure 4-1 [file zns999181219so1.tif]
