# Supplementary material for: Unilateral, 3D Arm Movement Kinematics Are Encoded in Ipsilateral Human Cortex
Source: J Neurosci. 2018 Nov 21;38(47):10042–56. doi: 10.1523/JNEUROSCI.0015-18.2018 (PMC6246886; doi:10.1523/JNEUROSCI.0015-18.2018)
Supplement: Figure 4-2 [file zns999181219so2.docx]

**Figure 4-2: Individual patient prediction statistics.** Full model predictions and surrogate predictions were compared for each individual patient using a rank-sum test. Median prediction accuracies, Wilcoxon rank-sum statistics (W), z-statistics, and p-values for each comparison are shown. Statistically significant differences after Bonferonni correcting for the total number of comparisons tested are highlighted in bold.

| **Contralateral** | **Actual** | **Temporal Surrogates** | | | **Feature Surrogates** | | |
| --- | --- | --- | --- | --- | --- | --- | --- |
|  | ***Median*** | ***Median*** | ***Effect Size*** | ***p*** | ***Median*** | ***Effect Size*** | ***p*** |
| *Speed (Pearson's r)* |  |  |  |  |  |  |  |
| Patient 1 | 0.3141 | 0.3068 | W(100,100)=10417, z=0.90 | 0.37 | 0.3053 | W(100,10000)=565072, z=2.07 | 0.039 |
| Patient 2 | 0.5881 | 0.2564 | **W(100,100)=14890, z=11.82** | **2.9x10^-32^** | 0.4276 | **W(100,10000)=949065, z=15.30** | **7.2x10^-53^** |
| Patient 3 | 0.7490 | 0.6736 | **W(100,100)=14462, z=10.78** | **4.3x10^-27^** | 0.6635 | **W(100,10000)=961329, z=15.73** | **9.9x10^-56^** |
| Patient 4 | 0.7961 | 0.6967 | **W(100,100)=14944, z=11.96** | **6.0x10^-33^** | 0.6905 | **W(100,10000)=995843, z=16.92** | **3.4x10^-64^** |
|  |  |  |  |  |  |  |  |
| *Vx (Pearson's r)* |  |  |  |  |  |  |  |
| Patient 1 | 0.3350 | -0.0349 | **W(100,100)=15033, z=12.17** | **4.3x10^-34^** | -0.0136 | **W(100,10000)=1003101, z=17.17** | **4.7x10^-66^** |
| Patient 2 | 0.3080 | -0.0105 | **W(100,100)=14144, z=10.0** | **1.5x10^-23^** | -0.0285 | **W(100,10000)=920651, z=14.32** | **1.5x10^-46^** |
| Patient 3 | 0.2086 | -0.0041 | **W(100,100)=14110, z=9.92** | **3.4x10^-23^** | 0.0184 | **W(100,10000)=891767, z=13.33** | **1.6x10^-40^** |
| Patient 4 | 0.6443 | -0.0446 | **W(100,100)=15048, z=12.21** | **2.7x10^-34^** | 0.0055 | **W(100,10000)=1004993, z=17.23** | **1.5x10^-66^** |
|  |  |  |  |  |  |  |  |
| *Vy (Pearson's r)* |  |  |  |  |  |  |  |
| Patient 1 | 0.4039 | 0.1076 | **W(100,100)=15044, z=12.20** | **3.1x10^-34^** | 0.0311 | **W(100,10000)=1004092, z=17.20** | **2.6x10^-66^** |
| Patient 2 | 0.2164 | 0.0512 | **W(100,100)=12833, z=6.80** | **1.1x10^-11^** | 0.0235 | **W(100,10000)=801602, z=10.22** | **1.6x10^-24^** |
| Patient 3 | 0.2530 | -0.0124 | **W(100,100)=14186, z=10.10** | **5.3x10^-24^** | 0.0486 | **W(100,10000)=894347, z=13.42** | **4.7x10^-41^** |
| Patient 4 | 0.4555 | 0.0030 | **W(100,100)=15016, z=12.13** | **7.1x10^-34^** | 0.0023 | **W(100,10000)=1001406, z=17.11** | **1.3x10^-65^** |
|  |  |  |  |  |  |  |  |
| *Vz (Pearson's r)* |  |  |  |  |  |  |  |
| Patient 1 | 0.1511 | -0.0056 | **W(100,100)=14556, z=11.01** | **3.5x10^-28^** | 0.0021 | **W(100,10000)=937034, z=14.89** | **3.9x10^-50^** |
| Patient 2 | 0.4627 | 0.1448 | **W(100,100)=14523, z=10.93** | **8.5x10^-28^** | 0.0574 | **W(100,10000)=966185, z=15.89** | **7.0x10^-57^** |
| Patient 3 | 0.2965 | -0.0193 | **W(100,100)=14642, z=11.22** | **3.3x10^-29^** | -0.0063 | **W(100,10000)=968684, z=15.98** | **1.8x10^-57^** |
| Patient 4 | 0.5737 | 0.0015 | **W(100,100)=15024, z=12.15** | **5.6x10^-34^** | -0.0167 | **W(100,10000)=1004559, z=17.22** | **2.0x10^-66^** |
|  |  |  |  |  |  |  |  |
| *Targets Hit (%)* |  |  |  |  |  |  |  |
| Patient 1 | 32.26% | 16.13% | **W(100,100)=14763, z=11.56** | **6.8x10^-31^** | 12.9% | **W(100,10000)=972828, z=16.30** | **9.2x10^-60^** |
| Patient 2 | 30.0% | 10.0% | **W(100,100)=13845, z=9.45** | **3.4x10^-21^** | 10.0% | **W(100,10000)=863619.5, z=12.94** | **2.6x10^-38^** |
| Patient 3 | 25.0% | 10.71% | **W(100,100)=13626, z=8.80** | **1.4x10^-18^** | 10.71% | **W(100,10000)=865101.5, z=12.60** | **2.0x10^-36^** |
| Patient 4 | 61.54% | 11.54% | **W(100,100)=15050, z=12.27** | **1.4x10^-34^** | 11.54% | **W(100,10000)=1005048, z=17.50** | **1.5x10^-68^** |
|  |  |  |  |  |  |  |  |
| **Ipsilateral** | **Actual** | **Temporal Surrogates** | | | **Feature Surrogates** | | |
|  | ***Median*** | ***Median*** | ***Effect Size*** | ***p*** | ***Median*** | ***W*** | ***p*** |
| *Speed (Pearson's r)* |  |  |  |  |  |  |  |
| Patient 1 | 0.3722 | 0.3628 | W(100,100)=10482, z=1.05 | 0.29 | 0.3621 | W(100,10000)=561992, z=1.96 | 0.05 |
| Patient 2 | 0.6355 | 0.5300 | **W(100,100)=14391, z=10.61** | **2.8x10^-26^** | 0.5271 | **W(100,10000)=934253, z=14.79** | **1.6x10^-49^** |
| Patient 3 | 0.7336 | 0.6216 | **W(100,100)=14859, z=11.75** | **7.1x10^-32^** | 0.6214 | **W(100,10000)=988186, z=16.65** | **2.9x10^-62^** |
| Patient 4 | 0.8103 | 0.7188 | **W(100,100)=14711, z=11.39** | **4.8x10^-30^** | 0.7130 | **W(100,10000)=976944, z=16.26** | **1.8x10^-59^** |
|  |  |  |  |  |  |  |  |
| *Vx (Pearson's r)* |  |  |  |  |  |  |  |
| Patient 1 | 0.3592 | 0.0272 | **W(100,100)=15025, z=12.15** | **5.4x10^-34^** | 0.0201 | **W(100,10000)=999308, z=17.04** | **4.5x10^-65^** |
| Patient 2 | 0.4135 | 0.1496 | **W(100,100)=14338, z=10.48** | **1.1x10^-25^** | -0.0147 | **W(100,10000)=990766, z=16.74** | **6.5x10^-63^** |
| Patient 3 | 0.4344 | 0.2902 | **W(100,100)=13466, z=8.35** | **7.1x10^-17^** | 0.1882 | **W(100,10000)=949168, z=15.31** | **6.8x10^-53^** |
| Patient 4 | 0.6899 | 0.0107 | **W(100,100)=15050, z=12.22** | **2.6x10^-34^** | 0.0208 | **W(100,10000)=1005049, z=17.23** | **1.5x10^-66^** |
|  |  |  |  |  |  |  |  |
| *Vy (Pearson's r)* |  |  |  |  |  |  |  |
| Patient 1 | 0.3338 | 0.0225 | **W(100,100)=14955, z=11.98** | **4.3x10^-33^** | 0.0372 | **W(100,10000)=991949, z=16.78** | **3.3x10^-63^** |
| Patient 2 | 0.1606 | 0.0307 | **W(100,100)=12789, z=6.69** | **2.2x10^-11^** | -0.0121 | **W(100,10000)=832739, z=11.29** | **1.4x10^-29^** |
| Patient 3 | 0.1683 | -0.0064 | **W(100,100)=13265, z=7.85** | **4.0x10^-15^** | 0.0152 | **W(100,10000)=812191, z=10.59** | **3.4x10^-26^** |
| Patient 4 | 0.5783 | -0.0104 | **W(100,100)=15043, z=12.20** | **3.2x10^-34^** | -0.0005 | **W(100,10000)=1004749, z=17.22** | **1.8x10^-66^** |
|  |  |  |  |  |  |  |  |
| *Vz (Pearson's r)* |  |  |  |  |  |  |  |
| Patient 1 | 0.2441 | 0.0161 | **W(100,100)=14177, z=10.08** | **6.6x10^-24^** | 0.0116 | **W(100,10000)=947893, z=15.26** | **1.3x10^-52^** |
| Patient 2 | 0.3306 | 0.1418 | **W(100,100)=13899, z=9.40** | **5.3x10^-21^** | 0.0468 | **W(100,10000)=956426, z=15.56** | **1.4x10^-54^** |
| Patient 3 | 0.2733 | 0.0874 | **W(100,100)=13368, z=8.11** | **5.2x10^-16^** | 0.0179 | **W(100,10000)=905290, z=13.80** | **2.7x10^-43^** |
| Patient 4 | 0.5118 | 0.0131 | **W(100,100)=15030, z=12.17** | **4.7x10^-34^** | 0.0191 | **W(100,10000)=1000045, z=17.06** | **2.9x10^-65^** |
|  |  |  |  |  |  |  |  |
| *Targets Hit (%)* |  |  |  |  |  |  |  |
| Patient 1 | 33.33% | 26.67% | **W(100,100)=11675, z=4.02** | **5.9x10^-9^** | 20.00% | **W(100,10000)=752809.5, z=8.68** | **4.0x10^-18^** |
| Patient 2 | 33.33% | 12.50% | **W(100,100)=14435, z=10.77** | **4.6x10^-27^** | 12.50% | **W(100,10000)=956313, z=15.84** | **1.6x10^-56^** |
| Patient 3 | 26.09% | 13.04% | **W(100,100)=13584, z=8.72** | **2.7x10^-18^** | 13.04% | **W(100,10000)=844041.5, z=11.89** | **1.3x10^-32^** |
| Patient 4 | 65.38% | 11.54% | **W(100,100)=15050, z=12.25** | **1.7x10^-34^** | 11.54% | **W(100,10000)=1005045.5, z=17.51** | **1.2x10^-68^** |
|  |  |  |  |  |  |  |  |
